# Validity and Reliability of the Newly Developed Malay-Language Health Belief of Bloating (HB-Bloat) Scale

**DOI:** 10.3390/ijerph17082773

**Published:** 2020-04-17

**Authors:** Nurzulaikha Abdullah, Yee Cheng Kueh, Garry Kuan, Mung Seong Wong, Fatan Hamamah Yahaya, Yeong Yeh Lee

**Affiliations:** 1 Unit of Biostatistics and Research Methodology, School of Medical Sciences, Universiti Sains Malaysia, Kelantan 16150, Malaysia; ngahpc@yahoo.com; 2 Exercise and Sport Science, School of Health Sciences, Universiti Sains Malaysia, Kelantan 16150, Malaysia; 3 Department of Life Sciences, Brunel University, London UB8 3PH, UK; 4 Department of Medicine, School of Medical Sciences, Universiti Sains Malaysia, Kelantan 16150, Malaysia; mswong@usm.my (M.S.W.); yylee@usm.my (Y.Y.L.); 5 School of Distance Education, Universiti Sains Malaysia, Pulau Pinang 11800, Malaysia; hamamah@usm.my; 6 Gut Research Group, Faculty of Medicine, Universiti Kebangsaan Malaysia, Kuala Lumpur 50300, Malaysia; 7 St George and Sutherland Clinical School, University of New South Wales, Sydney 2052, Australia

**Keywords:** abdominal bloating, questionnaire, theory of planned behavior, intention, self-management, lifestyle, quality of life

## Abstract

Abdominal bloating (AB), a common complaint that affects quality of life and disturbs psychological well-being, is largely a behavioral-driven disorder. We aimed to develop and validate a new health belief of bloating (HB-Bloat) scale in the Malay language. The initial item pool was developed based on the theory of planned behavior, empirical literatures, expert review and in-depth interviews. Using the population with bloating (diagnosed based on the Rome IV criteria and pictogram), exploratory and confirmatory factor analytical approaches (EFA and CFA, respectively) were utilized to explore and confirm the domains in the new scale. There were 150 and 323 respondents in the EFA and CFA, respectively. There were 45 items in the initial scale, but it was reduced to 32 items after content validity and pre-testing. In EFA, 17 items with three (3) structure factors (attitude 4 items, subjective norm 7 items, and perceived behavior control 6 items) were identified. Total variance explained by the EFA model was 40.92%. The Cronbach alpha of the three (3) factors ranged from 0.61 to 0.79. With CFA, the three factors model was further tested. Five problematic items were identified and removed. The final measurement model fit the data well (root mean square error of approximation (RMSEA (90% CI) = 0.054 (0.038, 0.070), Comparative Fit Index (CFI) = 0.941, Tucker–Lewis Fit Index (TLI) = 0.924, and standardized root mean squared residual (SRMR) = 0.044). The construct reliability of the final measurement model ranged from 0.76 to 0.84. As a conclusion, the new HB-Bloat scale is a valid and reliable tool for assessment of health beliefs in bloating.

## 1. Introduction

The Cambridge Dictionary defined bloating as “a condition in which the stomach swells and feels full and uncomfortable” [1]. The Rome foundation, in the Rome IV criteria, defined functional bloating as recurrent feelings of bloating or visible distention for at least three days per month, the onset of symptoms of at least six months prior to diagnosis, the presence of symptoms for at least three months, and an insufficient criteria of other functional gastrointestinal disorders, especially irritable bowel syndrome [2].

Abdominal bloating (AB) is an extremely common complaint in the population especially among Asians [3,4,5,6,7,8,9,10]. Approximately, 15−30% of the United States general population experienced AB [3,4], and it is among the most common of any symptoms reported in medical literature [5,6,7]. In the Asian population, comparable prevalence rates were about 15−23% [8], with a similar rate in Malaysia [9,10]. AB significantly affects the productivity and well-being or quality of life of affected individuals [7,11,12]. Psychological disturbances are also common in this condition, including anxiety and depression [13,14,15]. The condition is largely driven by inappropriate health beliefs, which can seriously affect an individual’s intention to treat AB, but also lead to misbehavior toward health intervention. The theory of planned behavior, incorporating the belief domain as one of the main aspects to be built as a construct, has been widely used by researchers in medical research [16,17,18,19,20].

Thus, it is essential to verify and quantify health misbeliefs, and this is best achieved through the means of a validated scale. However, we need to base the new scale on a model that will best fit bloating. One such model is the Theory of Planned Behavior (TPB), well-known for its ability to predict social and health behaviors of studied condition [21]. It is based on the manipulation of three core aspects in order to increase the chance of action or intention including attitude (favor of behavior), subjective norm (social pressure to act in a certain way) and perceived behavioral control (power that controls action) [22]. Behavior, the central core in the TPB model, acts on the principle of compatibility [23] instead of general behavior. Previous studies indicate that TPB helps in ensuring the compliance of patients with treatment guidelines [22].

Based on our literature search, we found limited questionnaires related to AB, that were mainly focused on measuring the severity of the symptoms and quality of life. The available health belief scale used in the medical and health settings was still limited, and it varied between different symptoms or diseases, and most of them are not suitable to measure health belief related to AB [19,23,24,25]. Therefore, it is necessary to develop a new scale to measure the health belief related to AB for use in future studies and medical intervention. Thus, our aim was to first develop a new health belief scale for bloating, based on TPB, and subsequently to validate this new tool using exploratory and confirmatory approaches in people with bloating complaints.

## 2. Materials and Methods

### 2.1. Study Design, Recruitment, and Sampling

This was a cross-sectional study conducted between May 2018 and October 2019. A total of 473 participants were recruited within the compound of the Hospital Universiti Sains Malaysia (USM), situated at the north-eastern region of Peninsular Malaysia. Purposive random samples of hospital visitors including family members, care-givers, friends or staff were screened for study eligibility. The inclusion criteria were a diagnosis of bloating, both sexes, aged 18 years and above and in the absence of any history of organic GI diseases (e.g., inflammatory bowel disease, GI infections and colorectal cancer). To be included in the study, participants would have satisfied the Rome IV criteria for bloating, and/or have at least experienced one episode of bloating based on answers to verbal questions including “have you ever experience bloating?” and/or using a pictogram (approval obtained from the ROME foundation). Exclusion criteria included history of past abdominal surgeries, the current use of drugs which either cause or worsen bloating (e.g., opiates), and the presence of major psychiatric illnesses (e.g., schizophrenia).

### 2.2. Ethical Approval

Ethical approval was obtained from the Human Research Ethics committee, USM [USMKK/PPP/JEPEM/17010012]. The study conformed to the guidelines of the International Declaration of Helsinki with written informed consent was obtained from each participant.

### 2.3. Development and Validation of New Bloating Instrument

#### 2.3.1. Development of HB-Bloat Scale

Based on the concept of TPB, the newly developed Malay language health belief scale of abdominal bloating (HB-Bloat) consisted of three (3) domains, i.e., attitude, subjective norm, perceived control towards self-management. A multi-phase questionnaire development method was used, including gathering relevant items or contents from literature reviews, contents input from experts in the field, (with experience in health psychology, psychometric, bloating, and questionnaire development), and in-depth interviews with 12 individuals with functional bloating (based on the Rome IV criteria). In the initial stage of development, HB-Bloat contained 45 items with three (3) hypothesized domains. The three hypothesized domains consisted of 15 items for each domain. All responses were measured on a five-point Likert scale from 1 (strongly disagree) to 5 (strongly agree).

#### 2.3.2. Content Validity and Pre-Testing of the HB-Bloat Scale

The initial HB-Bloat scale with 45 items was tested for content validity. Seven (7) experts who were experts in gastrointestinal field, psychometric testing, language, and questionnaire development were invited to rate and comment on the items. The content validation index (CVI) computed based on responses of relevancy from experts was used to examine the content validity of the scale [26,27,28,29]. The content validity of items (I-CVI) for HB-Bloat scale ranged from 0.75 to 1.00. The content validity of the scale (S-CVI) for the three expected domains (or factors) in the HB-Bloat scale ranged from 0.90 to 0.94. These computed CVIs were considered satisfactory, as determined by Lynn [27].

Subsequently, pre-testing was conducted among 30 participants with a diagnosis of bloating. The participants were asked to comment on the clarity and comprehensibility of the administered HB-Bloat scale. In addition, format and font size were modified based on suggestions from participants. Participants commented that some items were either confusing or redundant due to their similarity to other items. Therefore, the questionnaire with 45 items was re-assessed by the experts again. The first version of HB-Bloat scale was then trimmed down from 45 to 32 items. Pre-testing on the 32 items was conducted again among 10 participants with a diagnosis of bloating. We found the result of the pre-test to be good and no modification was necessary. In order to determine the validity and reliability of this draft of HB-Bloat scale, exploratory factor analysis (EFA) was first performed followed by confirmatory factor analysis (CFA). The items of the HB-Bloat scale are presented in the supplemental file.

#### 2.3.3. Other Contents of the Final Questionnaire

In addition to items related to the diagnosis of bloating (Section 2.2) and the newly developed 32-item of HB-Bloat scale (Section 2.3), the final self-administered questionnaire also included socio-demographic information such as sex, age, ethnicity, religion, height, weight, and history of other medical and surgical illnesses.

### 2.4. Data Analysis

EFA and CFA were performed using the Statistical Package for Social Sciences (SPSS) version 24.0 (IBM, Armonk, NY, USA) and Mplus 8 was expressed as mean and standard deviation (SD) for numerical items and frequency and % for categorical variables.

#### 2.4.1. Exploratory Factor Analysis (EFA)

A total of 150 participants enrolled for the EFA study. Principal axis component with Promax rotation was performed on the 32 completed items to extract the major contributing factors. The number of factors with an eigenvalue greater than one was further inspected. Those with factor loading greater than 0.40 were regarded as significantly relevant and were kept for further analysis [29,30]. Deletions of items were then performed, the factor loading re-examined and the EFA model re-specified following each deletion. For an acceptable internal consistency of each construct, a Cronbach’s alpha value of 0.60 or higher was considered acceptable [29].

#### 2.4.2. Confirmatory Factor Analysis (CFA)

A total of 323 participants enrolled for CFA study. We further tested the EFA-derived model with CFA. Based on the Mardia test, the multivariate skew (*p* < 0.001) and kurtosis (*p* < 0.001) normality assumption was violated. Thus, the robust maximum likelihood estimator (MLR) was used in the subsequent factor analyses. During the model re-specification process, items with factor loading less than 0.40 were removed iteratively. The modification index (MI), as suggested by Mplus, was inspected, and items’ error covariance were added if necessary. Items with a standardised residual value of more than 4.0 were inspected and removed. All re-specification of the model was performed after adequate theoretical support was carried out by researchers.

The fit indices of the model were assessed using a standardized root mean squared residual (SRMR) lower than 0.06 indicating a perfect fit [23], the root mean square error of approximation (RMSEA) less than 0.08 suggested a reasonably good fit [21], and relative fit indices like the Tucker–Lewis Fit Index (TLI) and Comparative Fit Index (CFI), where a cut-off point of 0.92 indicated an acceptable model fit [29,30,31,32,33].

Convergent validity was assessed through construct reliability (CR) with an acceptable value of 0.70 or higher, and average variance extracted (AVE) with an acceptable value of 0.50 or higher [29,30,31]. If the AVE value is less than 0.50, but the CR value is more than 0.60, the convergent validity is still considered acceptable [31]. An acceptable discriminant validity of a factor was determined through correlation between factors using Pearson correlation coefficient (r) values lower than 0.85 [29,31,33].

## 3. Results

### 3.1. Demographic Characteristics of Participants in EFA and CFA

For EFA, the 150 participants had a mean age of 31.27 years old (SD = 14.36) and most were females (68.3%), while for CFA, the 323 participants had a mean age was 27.69 years old (SD = 11.50) and more than half were males (59.4%). The results are summarized in Table 1. Both groups had a very similar mean BMI.

### 3.2. EFA Results of the HB-Bloat Scale

**Figure 1:**
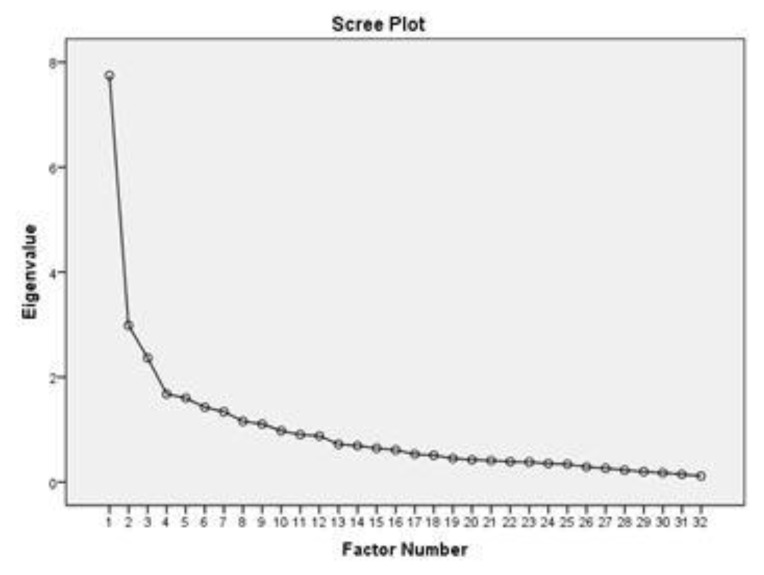
The Scree Plot of exploratory factor analysis (EFA).

The initial principle axis factoring analysis of all 32 items in HB-Bloat indicated sampling adequacy thus a reliable estimate from our current model. The computed Kaiser–Meyer–Olkin (KMO) value of 0.766 was considered good and the Bartlett’s test of sphericity was significant (*p* < 0.001), again supporting the validity of our EFA model. The items were run with EFA to explore the domain and were found to have nine domains with total variances of 54.26%. The Scree Plot is shown in Figure 1. We investigated the nine domains and their loaded items, and found that they did not fit into the theoretical construct of TPB. Therefore, the next step was proceeded by fixing the number of factors to three, as this construct used TPB as the theoretical support; parallel to that, it was suggested to be divided to three domains only. Then, the three (3) factors appeared to have eigenvalues above 1—indicative of acceptable importance—and cumulatively explained 40.9% of all responses. In addition, the rotated three factors model had more theoretical relevance to TPB. The variance value of each of three factors was 24.21, 9.32 and 7.39, respectively. The three (3) factors were “attitude,” “subjective norm,” and “perceived behavioral control” towards self-management (Table 2).

Several EFAs were performed sequentially and items deleted until all items factored above 0.40 with the absence of cross-loadings. Using this technique, 15 items were eventually deleted, and the remaining 17 items in the three domains (factors) were further analyzed with CFA.

Table 2 summarizes the results for descriptive statistics, EFA, and internal consistency (Cronbach’s α coefficient). The three extracted factors represented 40.92% of the variance in the 17 items. These were “attitude,” “subjective norm,” and “perceived behavioral control” towards self-management of bloating based on TPB. The reported Cronbach’s alpha values ranging from 0.61 to 0.79 were obtained for each subscale and showed good reliabilities of domains in the scale.

### 3.3. CFA Results of the HB-Bloat Scale

As shown in Table 3, the CFA of 17-item 3-factor EFA-model revealed that fit indices for Model−1 were not within acceptable threshold values except for RMSEA and SRMR. To improve the fit indices, MI value and standardized residual were inspected. There were five problematic items (i.e., A12, SN4, SN7, PBC1, PBC8) with standardized residual values higher than 4.0. After discussion among the researchers, several model re-specifications were conducted interactively by deleting problematic items from the measurement model, resulting in models 2. After re-specifications, the re-computed fit indices were much improved and the final measurement model (Model-2) is presented in Table 3.

Table 4 shows all the standardized factor loadings that exceeded the threshold value of 0.40. The CR values for all three factors were greater than 0.70, which indicated acceptable constructs reliability. Although the AVE values for the three factors were below the recommended value of 0.50, the convergent validity of the construct was considered adequate with all CR values above 0.60.

### 3.4. Discriminant Validity of the HB-Bloat Scale

Table 5 shows correlation between factors. The correlations were low to moderate and below the value of 0.85. The results supported good discriminant validity for domains in the new scale.

## 4. Discussion

In this study, we found that the newly developed 12-items 3-domains Malay-language HB-Bloat Scale is valid and reliable—evidenced by its superior psychometric properties. Furthermore, the new scale demonstrated good construct validity with all items’ loading more than 0.40 in EFA (except one item but was kept for further analysis based on suggestion from expert) and CFA. The reliabilities based on Cronbach alpha ranging from 0.61 to 0.79 were considered acceptable [29]. The HB-Bloat Scale will benefit patients with bloating, a common condition in clinical practice that is driven largely by behavioral dysfunction.

Evidence indicates that abdominal bloating may be modifiable or preventable through lifestyle changes [34]. Health behavior and behavioral change are important determinants in lifestyle manipulation strategy, and both often operate simultaneously. Various theories have been introduced for health behavior and psychology including the Health Beliefs Model (HBM; [35], Theory of Planned Behavior (TPB; [21]), Theory of Reasoned Action (TRA; [36]), and Pender’s Health Promotion Model (PHP; [37]). Among these theories, TPB is probably the preferred approach for a condition like bloating, and previous studies have proven that TPB works [18,21,38,39,40].

Thus far, there is no single tool or diagnostic marker that can reliably identify bloating. Often, clinicians and researchers would rely on a set of questions including the Rome IV criteria or the pictogram to diagnose bloating. However, no questionnaire exists that addresses the behavioral and psychological perception of bloating—thus the current study is important. The newly developed HB-Bloat Scale can fill this important gap. In order to achieve this, the new scale must be evidence-based but also relevant in real-life conditions. Therefore, we developed the HB-Bloat Scale based on an extensive review of literature, experts’ reviews and qualitative approaches through in-depth interviews until saturation. It was developed in the Malay language to suit the population we had studied. The Malay language is of Austronesian origin and is currently the fourth most widely spoken language in the world. It is the native language of more than 200 million people, largely residing in the South-East Asian countries including Malaysia [41,42].

Once we have a scale, we need to prove its validity and reliability. This can be achieved using EFA and CFA approaches. The EFA results firstly identified three (3) main factors, i.e., “attitude or A,” “subjective norm or SN,” and “perceived behavioral control or PBC” to self-management, and these derived beliefs were in parallel with our hypothesized model based on TPB. All items were gathered from the multi-phase questionnaire development process and finalized into 45 items after being reviewed by the experts. The main reason for having a large number of items (15 items per theoretical domain) in the initial stage is to be prepared that there would be some problematic items during the construct validity phase, as suggested in other studies [43,44]. Since this is a new questionnaire, we preferred to have more suitable items in each domain in order to explore and to confirm the best items that fit the model and data. The CFA results validated the final 17-item, 3-factor model of our new scale, with the elimination of other poor performing items in the initial 32-item EFA stage. The good loading items in the CFA results demonstrated coherence between the data and the structure based on fit indices criteria [26,28,33,45]. The final CFA model—which covered three (3) A items, five (5) SN items, and four (4) PBC items—showed an adequate model fit as the three-factor model fit the data well based on most of the fit indices shown in the present study. All domains had acceptable internal consistency and discriminant validity. Overall, this scale has a good theoretical basis and is ready for application in real practice. The overall intention of the HB-Bloat scale is to detect bloating misbeliefs and hopefully this may trigger self-intention towards individual management of the condition.

There were some limitations identified in the present study. The present study used a different set of samples for EFA and CFA phases. The participants were randomly selected from the study population, which explained the difference in proportions of sociodemographics between the two phases. The sample size for each phase was calculated accordingly for EFA and CFA. Because of some logistic issues and time constraint, this study incorporated a thorough multi-phase development stage with the internal validation of EFA and CFA without collecting other, different types of validity evidence that include the application of other similar scales or evidence of their predictive validity. Hence, future studies should include other types of validity evidence testing on the BH-Bloat scale. Although we have developed the first structural tool to assess the health beliefs of bloating, it is limited to the population and language studied. We suggest that the newly developed questionnaire needs to be translated into other languages, and to validate it with other populations, and with different cultures. Multi-group CFA tests can be another alternative test to examine the comprehensibility and stability of the new HB-Bloat scale.

## 5. Conclusions

The ability to assess the health beliefs of bloating is important to drive self-management of patients with this condition. The newly developed BH-Bloat scale, with 12 items and 3 domains, is shown to be valid and reliable in the current study. Further studies are needed in different population settings and languages.

## Figures and Tables

**Table 1:** Demographic characteristics for participants in EFA and CFA.

Variables	EFA (150)		CFA (323)	
	Mean (SD)	*n* (%)	Mean (SD)	*n* (%)
Age	31.27 (14.36)		27.69 (11.50)	
Weight	62.42 (12.63)		62.09 (13.36)	
Height	158.90 (7.08)		160.48 (11.79)	
BMI	24.79 (4.52)		24.90 (14.20)	
Gender				
Male		35 (24.6)		192 (59.4)
Female		97 (68.3)		114 (35.3)
No response		18 (7.1)		17 (5.3)
Ethnics				
Malay		127 (89.4)		291 (90.1)
others		4 (2.8)		16 (5.0)
No response		19 (12.7)		16 (4.9)
Address				
Rural		76 (53.5)		170 (52.6)
Urban		43 (30.3)		136 (42.1)
No response		31 (20.7)		17 (5.3)
Other symptoms				
No		93 (65.5)		264 (81.7)
Yes		31 (21.8)		49 (15.2)
No response		26 (17.3)		10 (3.4)
Other disease				
No		107 (75.4)		264 (88.9)
Yes		16 (11.3)		23 (7.1)
No response		27 (13.3)		36 (11.1)

**Table 2:** Results of Descriptive Statistics, Exploratory Factor Analysis, and Reliability Analysis for EFA Sample (*n* = 150).

No. Abbreviated Item Content	Mean	SD	FACTOR LOADING		
			1	2	3
A1	4.44	0.54	0.724		
A2	3.82	0.84	-		
A3	3.96	0.71	-		
A4	4.09	0.47	-		
A5	4.21	0.69	-		
A6	3.94	0.69	-		
A7	4.22	0.61	0.797		
A8	3.90	0.66	-		
A9	4.11	0.54	-		
A10	4.19	0.75	0.699		
A11	4.10	0.61	-		
A12	4.31	0.63	0.495		
A13	3.98	0.45	-		
SN1	3.80	0.66		-	
SN2	3.84	0.70		0.636	
SN3	3.78	0.99		0.612	
SN4	3.98	0.66		0.735	
SN5	3.77	0.74		0.758	
SN6	3.87	0.73		0.646	
SN7	4.20	0.62		0.596	
SN8	3.70	0.84		0.635	
PBC 1	4.00	0.58			0.409
PBC 2	3.82	0.93			-
PBC 3	4.19	0.51			-
PBC 4	4.17	0.65			-
PBC 5	4.17	0.62			0.697
PBC 6	4.00	0.53			0.743
PBC 7	3.92	0.49			0.743
PBC 8	3.72	0.70			0.649
PBC 9	4.21	0.46			0.330
PBC 10	4.21	0.70			-
PBC 11	4.12	0.51			-
Eigenvalue			7.75	2.98	2.36
Variance explained (%)			24.21	9.33	7.39
Cumulative variance (%)			24.21	33.54	40.92
Cronbach alpha			0.71	0.79	0.61

Legend: A (attitude domain), SN (subjective norm) and PBC (perceived behavioral control).

**Table 3:** Summary for HB-Bloat-M model fit indices (*n* = 323).

Path Model	RMSEA (90% CI)	CFI	TLI	SRMR
Model-1	0.069 (0.059, 0.078)	0.877	0.856	0.059
Model-2 ^a^	0.054 (0.038, 0.070)	0.941	0.924	0.044

^a^ Model-2 with reduced items, A12, SN4, SN7, PBC1, PBC8.

**Table 4:** Standardised Factor Loading of Confirmatory Factor Analysis for the CFA Sample (*n* = 323).

Constructs/Items	Mean	SD		Model 1			Model 2	
			λ	AVE	CR	λ	AVE	CR
**Attitude**				0.44	0.75		0.44	0.70
A1	4.28	0.68	0.67			0.64		
A7	4.21	0.69	0.68			0.73		
A10	4.06	0.82	0.57			0.62		
A12	4.23	0.67	0.71			-		
**Subjective norm**				0.42	0.84		0.42	0.78
SN2	4.12	0.77	0.70			0.68		
SN3	4.02	0.76	0.57			0.62		
SN4	4.22	0.68	0.66			-		
SN5	4.04	0.71	0.64			0.65		
SN6	4.11	0.72	0.68			0.70		
SN7	4.13	0.68	0.75			-		
SN8	4.16	0.71	0.54			0.59		
**Perceived behavioral control**				0.39	0.79		0.42	0.75
PBC1	4.26	0.61	0.63			-		
PBC5	4.23	0.67	0.64			0.62		
PBC6	4.16	0.74	0.68			0.70		
PBC7	4.07	0.76	0.62			0.63		
PBC8	4.09	0.69				-		
PBC9	4.33	0.60	0.65			0.65		

Note: λ = standardized factor loading, CR = construct reliability, AVE = average variance extracted, all factor loadings were statically significant at *p* < 0.050.

**Table 5:** Discriminant Validity among Latent Variables of Confirmatory Factor Analysis for the Validation Sample (*n* = 323).

Constructs/Pearson Correlation Coefficient, r	1	2	3
ATT	1	0.67	0.71
SN		1	0.69
PBC			1

Note: all correlation coefficients were statistically significant at *p* < 0.050.

## Supplementary Materials

Supplementary materials can be found at https://www.mdpi.com/1660-4601/17/8/2773/s1.
